# Enteric infection coupled with chronic Notch pathway inhibition alters colonic mucus composition leading to dysbiosis, barrier disruption and colitis

**DOI:** 10.1371/journal.pone.0206701

**Published:** 2018-11-01

**Authors:** Ishfaq Ahmed, Badal C. Roy, Rita-Marie T. Raach, Sarah M. Owens, Lijun Xia, Shrikant Anant, Venkatesh Sampath, Shahid Umar

**Affiliations:** 1 Department of Surgery, University of Kansas Medical Center, Kansas City, Kansas, United States of America; 2 Department of Molecular Biosciences, University of Kansas, Lawrence, Kansas, United States of America; 3 Biosciences Division, Argonne National Laboratory, Lemont, Illinois, United States of America; 4 Oklahoma Medical Research Foundation, University of Oklahoma Health Sciences Center Oklahoma City, Oklahoma, United States of America; 5 Division of Neonatology, Children’s Mercy Hospital, Kansas City, Missouri, United States of America; Cincinnati Children's Hospital Medical Center, UNITED STATES

## Abstract

Intestinal mucus layer disruption and gut microflora modification in conjunction with tight junction (TJ) changes can increase colonic permeability that allows bacterial dissemination and intestinal and systemic disease. We showed previously that *Citrobacter rodentium* (CR)-induced colonic crypt hyperplasia and/or colitis is regulated by a functional cross-talk between the Notch and Wnt/β-catenin pathways. In the current study, mucus analysis in the colons of CR-infected (10^8^ CFUs) and Notch blocker Dibenzazepine (DBZ, i.p.; 10μmol/Kg b.w.)-treated mice revealed significant alterations in the composition of trace *O*-glycans and complex type and hybrid *N*-glycans, compared to CR-infected mice alone that preceded/accompanied alterations in 16S rDNA microbial community structure and elevated EUB338 staining. While mucin-degrading bacterium, *Akkermansia muciniphila* (*A*. *muciniphila*) along with *Enterobacteriaceae* belonging to *Proteobacteria* phyla increased in the feces, antimicrobial peptides Angiogenin-4, Intelectin-1 and Intelectin-2, and ISC marker Dclk1, exhibited dramatic decreases in the colons of CR-infected/DBZ-treated mice. Also evident was a loss of TJ and adherens junction protein immuno-staining within the colonic crypts that negatively impacted paracellular barrier. These changes coincided with the loss of Notch signaling and exacerbation of mucosal injury. In response to a cocktail of antibiotics (Metronidazole/ciprofloxacin) for 10 days, there was increased survival that coincided with: i) decreased levels of *Proteobacteria*, ii) elevated Dclk1 levels in the crypt and, iii) reduced paracellular permeability. Thus, enteric infections that interfere with Notch activity may promote mucosal dysbiosis that is preceded by changes in mucus composition. Controlled use of antibiotics seems to alleviate gut dysbiosis but may be insufficient to promote colonic crypt regeneration.

## Introduction

The gastrointestinal tract is a complex ecosystem that encounters food, microorganisms, foreign antigens, and toxic molecules on a daily basis. It is therefore pivotal for the endogenous barrier mechanisms to limit the exposure of epithelial monolayers to intraluminal entities in order to maintain gut immune homeostasis. Colonic mucus layer comprises the first line of defense and prevents entry of bacteria while allowing diffusion of essential nutrients [1, 2]. Mucins constitute the structural and functional units of the mucus layer in human beings and mice, with Muc-2 representing the principal component of colonic mucus [3, 4]. Muc-2 is composed primarily of *O*-glycans, which have two main subtypes known as Core-1 and Core-3-derived *O*-glycans [5, 6]. Due to a high degree of glycosylation, mucins provide growth substrate and site of adhesion for mucus-associated microbiota [7], that when healthy, exist in a symbiotic relationship with the host [8]. The intestinal microbiota modulates a variety of host responses including those related to metabolism, which the host has not developed for itself [8–10]. The microbiota provides an energy source to colonocytes in the form of short-chain fatty acids (SCFAs) through scavenging of carbohydrates from both dietary sources and colonic mucins [7]. Microbiota resists and competes with pathogens for space and resources, elaborates the molecules required for mucosal integrity and modulates the immunological responses [11].

Below the mucus layer, there is a monolayer of epithelial cells that are held together by tight and adherens junctions (TJs/AJs) and characterized by a remarkable polarization of their plasma membrane that restricts both transcellular and paracellular permeation of antigens, allowing only limited quantities of molecules to cross into the mucosa in a controlled manner. The sustained enhancement of paracellular permeability facilitates the constant passage of luminal antigens through the mucosa which can lead to interaction with the mucosal immune system and chronic inflammation in susceptible individuals [12].

In the colon, Notch activation modulates expression of Muc-2 and TJ proteins and the balance between proliferation and differentiation in the enterocyte progenitor pool [13, 14]. Following inhibition of Notch signaling, there is an exit from the proliferation compartment and differentiation into the post-mitotic goblet cells [15]. We showed previously in a murine model of *Citrobacter rodentium (CR)*-induced colonic crypt hyperplasia that chronic inhibition of Notch signaling results in severe inflammation, morbidity and mortality in an outbred strain that otherwise exhibits a self-limiting disease [16]. In the current study, we tested the hypothesis that CR infection combined with chronic Notch pathway inhibition may alter the composition of the colonic mucus and the ensuing dysbiosis, tight junction disruption and loss of multipotent intestinal stem cells (ISCs) may facilitate colitis-like disease development.

## Materials and methods

### Animals

NIH:Swiss outbred and C3H/HeNHsd (C3H) inbred mice were procured from Harlan Laboratories Inc. USA. C3H mice respond very aggressively to CR infection and are used as an excellent model of infectious colitis [17]. *Core-3*^*-/-*^ mice lacking Core-3 β1,3-N-acetylglucosaminyltransferase (C3GnT), an enzyme predicted to be important in the synthesis of Core 3-derived O-glycans, against a mixed background strain of C57BL/6J were generated as described [5]. *Rag-1*^*-/-*^ mice lacking mature B and T-cells (Stock # 002216) in the C57BL/6J background were purchased from Jackson Laboratory. All the mice were maintained in a specific pathogen-free (including Helicobacter and parvovirus) environment and generally used between 5 and 6 weeks of age. As control groups, either littermates or WT mice of identical background were used. This study was carried out in strict accordance with the recommendations in the Guide for the Care and Use of Laboratory Animals of the National Institutes of Health. All protocols were approved by the University of Kansas Medical Center Animal Care and Use Committee.

### Treatments

Transmissible Murine Colonic Hyperplasia was induced in the mice by oral inoculation with a 16-h culture of *C*. *rodentium* (biotype 4280, ATCC, 10^8^CFUs) identified as pink colonies on MacConkey agar, as previously described [17–25]. Biotype 4280 is a unique mouse-specific strain that adheres to mature surface colonocytes within the distal colon to induce histopathological changes known as attaching and effacing lesions [26]. Adherent bacteria were assayed using RT-PCR for bacterial intimin in whole tissue extracts [23]. Age- and sex-matched control mice received sterile culture medium only.

To block Notch signaling *in vivo*, we used a cell-permeable inhibitor of γ-secretase, Dibenzazepine (DBZ) [27] (EMD Chemicals, Inc., Gibbstown, USA). DBZ was suspended finely in 0.5% (w/v) HPMC and 0.1% (w/v) Tween-80 in water and given to mice intraperitoneally (at 10μmol/kg body weight) for 10 consecutive days beginning 2 days post-CR infection. For depletion of microbiota, mice were given drinking water containing a combination of 1g/l metronidazole and 0.2 g/l ciprofloxacin (Wako) for 10 days.

### Histology, immunohistochemistry, and immunofluorescence

Colon tissues were freshly harvested from mice and fixed with 10% neutral buffered formalin or in Carnoy’s fixative (60% methanol, 30% chloroform, and 10% acetic acid) prior to paraffin embedding. Paraffin-embedded sections (4 μm) were stained with Hematoxylin and Eosin for morphology and with appropriate antibodies using standard techniques [28, 29]. Goblet cells were stained with PAS (Richard -Allan Scientific) or Alcian blue (Thermo-Scientific) and counterstained with Nuclear Fast Red (Sigma-Aldrich). The pictures were obtained with a Nikon i80 microscope.

### Fluorescence in-situ hybridization (FISH)

FISH was performed according to the method described previously with some modification [3]. Paraffin sections were dewaxed and rehydrated in an ethanol gradient to water. The tissue sections were incubated with 5 μg/ml TexasRed-conjugated EUB338 (5’- GCTGCCTCCCGTAGGAGT-3’, Invitrogen) in hybridization buffer (0.1M Tris-HCl, 0.9 M NaCl, 0.1% SDS and 10% formamide, pH 7.2) at 40°C overnight. The sections were rinsed in washing buffer (20 mM Tris-HCl, 0.9 M NaCl, pH 7.4) at 40°C for 15 min and stained with 1 μg/ml DAPI. After staining, the sections were mounted with Prolong Gold mounting medium (Invitrogen). All images were obtained and analyzed with a Nikon i80 microscope.

### Quantitative reverse-transcriptase PCR and Western blotting

RNA was isolated using TRIzol (Ambion Life Technologies) and converted to cDNA using the High-Capacity cDNA Reverse Transcription kit (Applied Biosystems). The concentration of RNA was measured using a spectrophotometer (Nanodrop 2000, Thermo-Scientific). Gene expression was assessed using Jumpstart Taq Polymerase (Sigma-Aldrich) and SYBR Green nucleic acid stain (Life Technologies). Threshold crossing values for each gene were normalized to GAPDH and mRNA expression was normalized to fold change relative to controls. Total crypt cellular or nuclear extracts (30–50 μg protein/lane), were subjected to SDS-PAGE and electrotransferred to nitrocellulose membrane. The membranes were blocked with 5% BSA or 5% nonfat dried milk in Tris-buffered saline (TBS) (20 mM Tris-HCl and 137 mM NaCl, pH 7.5) for 1 h at room temperature (21°C). Immunoantigenicity was detected by incubating the membranes overnight with the appropriate primary antibodies (0.5–1.0 μg/ml in 5% BSA or 5% nonfat dried milk). After washing, membranes were incubated with horseradish peroxidase-conjugated anti-goat, anti-mouse or anti-rabbit secondary antibodies and developed using the ECL detection system (GE) according to the manufacturer’s instructions.

### Bacterial DNA extraction and microbial analysis using 16S ribosomal DNA library preparation and sequencing

Fresh feces were collected in sterile tubes on ice and stored at −80°C until processing. Total genomic bacterial DNA was extracted using the QIAmp DNA stool kit (Qiagen, Valencia, CA) following their instructions. The integrity, concentration, and quality of the total DNA were assessed by agarose gel electrophoresis, and determined by absorption at A260, and A260 to A280 ratio, respectively using a Nanodrop-2000 spectrophotometer (Thermo Scientific Inc, USA). DNA solutions were stored at −20°C until further analysis. Using bacterial DNA, the V4 region of the 16S ribosomal RNA (rRNA) encoding gene was amplified with barcoded universal bacterial primers followed by sequencing on Ilumina MiSeq platform [30]. The resulting raw sequence files (.fastq.gz) were submitted to the NCBI Sequence Read Archive (SRA) database (https://www.ncbi.nlm.nih.gov/sra/SRP160909). The raw sequences were analyzed using open-source bioinformatics pipeline called Quantitative Insights Into Microbial Ecology (QIIME) [31]. Reads were trimmed and demultiplexed using exact matches to the supplied DNA barcodes. Any reads with homopolymer runs, more than 6 ambiguous bases, nonmatching barcodes, barcode errors, or quality scores less than 25 were removed. Samples with less than 3500 a sequence were also removed. Resulting sequences were searched against the Greengenes 13_5 reference sequence set and clustered at 97% by Uclust [32]. The centroid of each Operational Taxonomic Unit (OTU) was considered as the OTU representative sequence followed by aligning the sequences with PyNast and construction of Trees with FastTree for phylogenetic calculations [33].

### Fluorescein isothiocyanate-dextran (FITC-D) assay

*In-vivo* permeability assay to assess epithelial barrier function was performed using FITC-D as described [34]. Briefly, food was withdrawn for 4 h from 5- to 6-weeks-old NIH: Swiss mice in various groups and gavaged with 80mg/100g body weight of FITC-D, (molecular weight 4,000; Sigma-Aldrich). Serum was collected at the time of euthanasia and the fluorescence intensity of each sample was measured with a fluorimeter (excitation, 492 nm; emission, 525 nm; FLUOstar Galaxy 2300; BMG Labtech, Durham, NC). FITC-D concentrations were determined from standard curves generated by serial dilution of FITC-D and permeability was calculated by linear regression of sample fluorescence (Excel 5.0; Microsoft).

### Carbohydrate analysis

NIH:Swiss mice were euthanized and mucosal scrapings of the distal colon were collected with EDTA free protease inhibitor (Roche). Samples of colon mucus were analyzed for *O*-type and *N*-type glycosylation at Complex Carbohydrate Research Center, Athens, GA. Samples were depleted of lipids, proteins were precipitated and the protein-rich powder was treated with trypsin, purified and finally treated with PNGase F to remove *N*-linked glycans. Following removal of *N*-glycans, the *O*-glycans were removed through beta-elimination procedures and both the *N*- and *O*-linked glycans were analyzed through standard techniques [35].

### Statistical analyses

Experiments were repeated three times with consistent results. Data were expressed as mean values ± standard error. Statistical analyses for all studies were performed using unpaired, two-tailed Student’s *t*-tests and one-way analysis of variance (ANOVA) for multiple group comparisons (GraphPad Prism 5, San Diego, CA). *p*-values < 0.05 were considered statistically significant.

## Results

### Alteration of mucus glycans and bacterial dysbiosis

**Fig 1:**
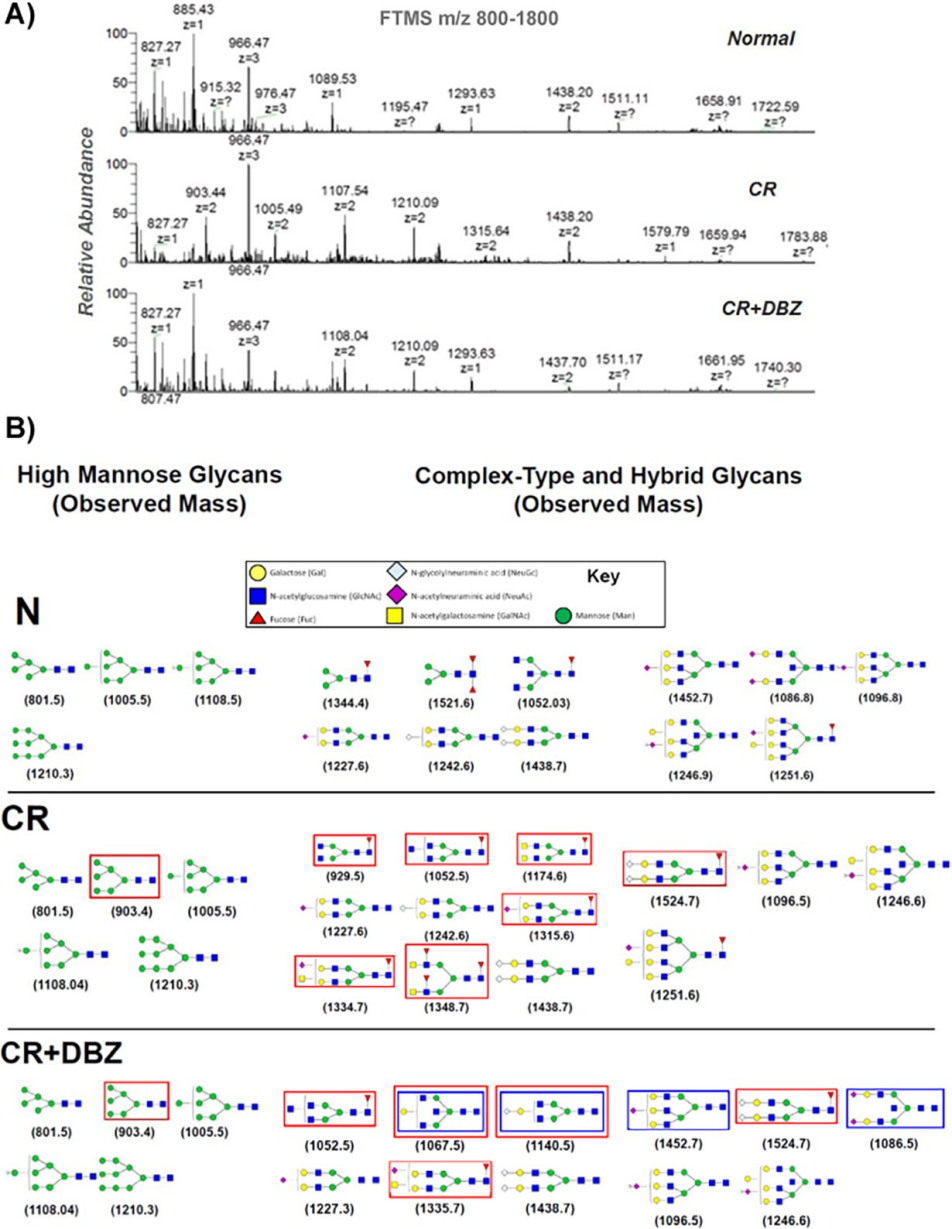
Analysis of colon mucus samples for *N*-type glycosylation. **A. FTMS m/z 800–1800 of mucus samples**. Processing of mucus samples from uninfected (Normal), CR-infected (CR) and CR+DBZ-treated (CR+DBZ) mouse colons included removal of lipids and precipitation of proteins into a protein-rich powder that was subsequently treated with trypsin, purified, then treated with PNGase F, to remove *N*-linked glycans. A full FTMS spectrum was collected at 30,000 resolution with 3 microscans. The highest abundance glycans are primarily high mannose, and complex-type species containing *N*-glycolylneuraminic acid (NeuGc). MS/MS analysis of the highest abundance complex-type glycans appears as a z = 3 ion at m/z 966. **B. Detection of glycans from each sample, as well as the results of each MS/MS analysis from total ion mapping (TIM) analysis.** Some representative mucus glycan structures are shown together with the terminal end structures including the interconnecting linkages of the different monosaccharides, which are explained in the key. Red rectangles represent differences between uninfected (N) and CR-infected (CR) mucus samples while blue rectangles represent mucus glycans unique to CR+DBZ-treated mice.

**Fig 2:**
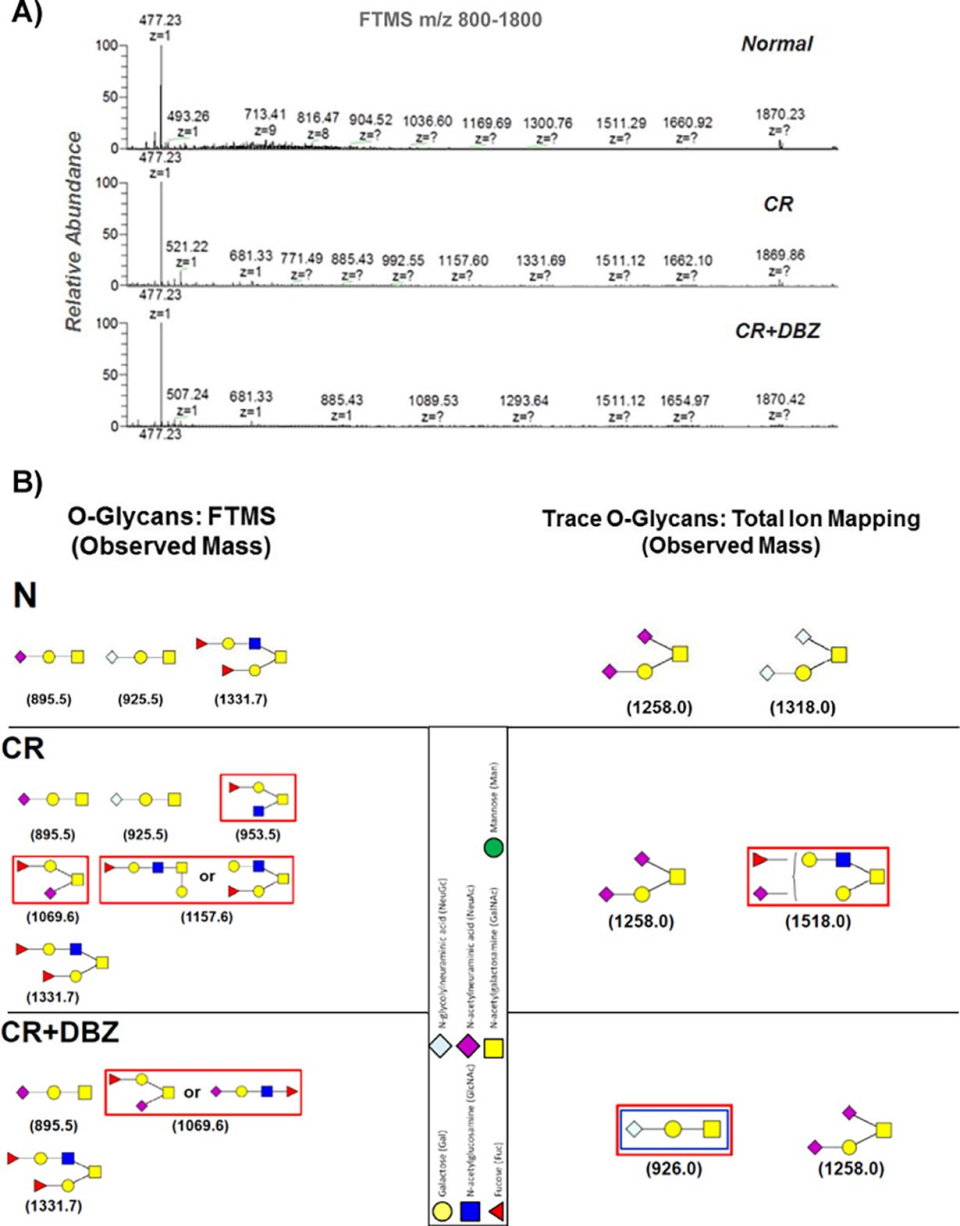
Analysis of colon mucus samples for *O*-linked glycans. **A. FTMS m/z 800–1800 of mucus samples.** After removing the N-glycans from the proteins with PNGase F, the *O*-glycans were removed through beta-elimination procedures. The glycans were then purified again with a C18 column, permethylated, purified again, then analyzed with MALDI and NSI-MS. Numerous *O*-glycans are detected in the samples. Additionally, a number of trace glycans are detected as a result of Total Ion Mapping (TIM). **B. *O*-Glycans Detected by FTMS.** Representative *O*- structures assigned are “proposed” structures based on the fall and tandem MS data observed, as well as common biosynthetic pathways common to mammalian and (if possible) the species *Mus Musculus*. Red rectangles represent differences between normal (N) and CR-infected (CR) mucus samples while blue rectangles represent mucus glycans unique to CR+DBZ-treated mice.

**Fig 3:**
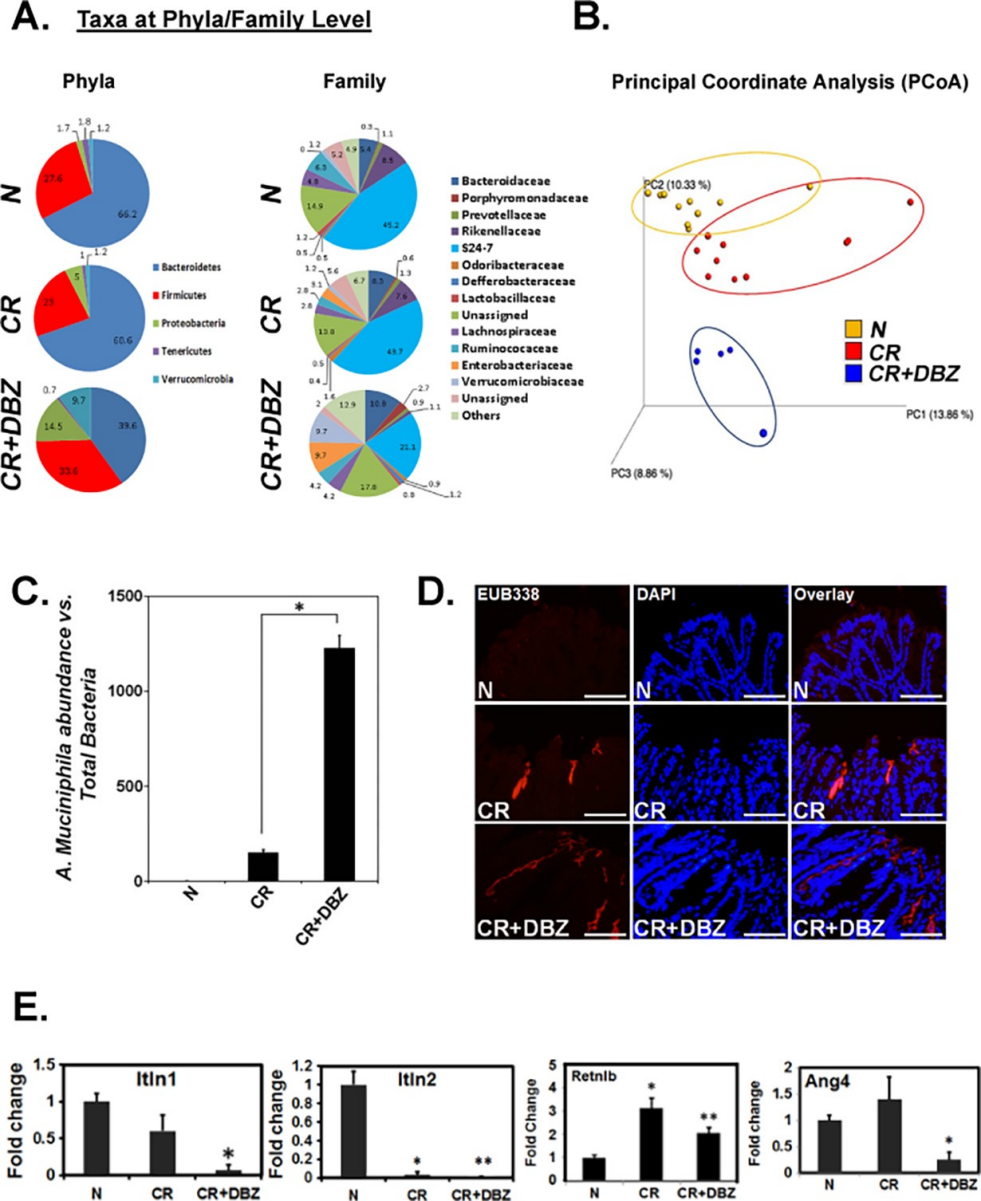
Evidence of bacterial dysbiosis during chronic blockade of Notch pathway in the colons of outbred mice. **A. Comparison of major microbial populations in fecal samples.** Fecal samples from uninfected (N), CR-infected (CR) and CR+DBZ-treated mice were subjected to 16S rDNA sequencing and relative abundance of phyla and families were compared. Each chart represents the taxonomic composition in the indicated groups (n = 10 mice/group). **B. Principal Coordinate Analysis (PCoA) of fecal microbiome composition.** While N (shown in “orange”) and CR (shown in “red”) samples grouped closer to each other, CR+DBZ samples (shown in “blue”) shifted towards the opposite ends of the coordinates revealing distinct microbial communities (p<0.001; n = 10 mice/group). **C. Species identification as a potential etiologic agent.** Real-time qPCR showing relative abundance for *A*. *mucinophila* in various treatment groups (p<0.005; n = 3 independent experiments). **D. Fluorescence microscopy to detect bacterial invasion**. The attached bacteria in the flushed colonic tissues of N, CR or CR+DBZ mice were detected by FISH using a general bacterial 16S probe (TexasRed-Eub338; Bar = 100μm; n = 10 mice/group). DAPI was used as counter-stain. **E. Effect of chronic Notch inhibition on anti-bacterial peptide gene expression.** qPCR to examine expression levels of antibacterial peptide genes *Itln1*/*2*, *Retnlb* and *Ang4* encoding Intelectin-1/2, Resistin-like molecule-β and Angiogenin-4, respectively (*, **p<0.005; n = 3 independent experiments).

We have shown previously that CR infection coupled with Notch pathway blockade promotes goblet cell hyperplasia and colitis [16]. To determine why despite mucus hypersecretion, mice endured barrier disruption and severe inflammation, we tested the hypothesis that compositional changes in mucus glycans may lead to bacterial dysbiosis and facilitate the development of the colitis-like disease. Figs 1 and 2 reveal the base peak chromatograms of *O*- and *N*-glycans from these mice wherein marked differences were evident between uninfected, CR-infected and CR+DBZ groups. In particular, altered glycosylation pattern in CR+DBZ group consisting of high mannose and complex-type and hybrid glycans and accompanied by loss of fucosylated *N*-glycans compared with CR infected alone strengthens the hypothesis that chronically blocking Notch signaling alters the mucus composition. Since the resulting changes in glycosylation may affect the supply of carbohydrates available to lumenal bacteria utilizing mucin glycans as carbon source thereby changing the microbiota composition, we next performed 16S rRNA gene sequencing. We discovered that as compared to CR, CR+DBZ group showed a significant decrease in *Bacteroidetes* phyla with concomitant increases in *Firmicutes*, *Proteobacteria* and *Verrucomicrobia* phyla respectively. In particular, we discovered *Enterobacteriaceae* and *Verrucomicrobiacea* families to be over-represented in the CR+DBZ group (Fig 3A). Principal coordinate analysis (PCoA) revealed a significant separation of microbial communities in fecal samples from CR+DBZ mice when compared to either uninfected or CR-infected mice (p-value = 0.001) (Fig 3B). Comparison of sequences at species level showed that relative abundance of mucin-degrading bacterium *A*. *muciniphila* belonging to the *Verrucomicrobia* phyla [36] was elevated in the CR+DBZ mice than either uninfected or CR-infected mice (Data not shown) that coincided with mucus layer disruption as was shown by us previously [16], suggesting that mucus degradation may precede onset of colitis in CR+DBZ-treated mice. An increase in *A*. *muciniphila* in CR+DBZ compared to N and CR was validated by qPCR (Fig 3C). We next assessed bacterial invasion into the colonic epithelium in the CR+DBZ group by FISH using a ubiquitous eubacterial probe, EUB338. As is revealed in Fig 3D, significantly more bacteria colonized the mucosa while only a small number of bacteria invaded the colonic crypts in response to CR infection. In the CR+DBZ group, however, a dramatic increase in bacterial colonization of the crypts was observed (Fig 3D). To explore if the loss of antibacterial peptide gene expression may have led to higher bacterial burden in the CR+DBZ mice, we next looked at the expression levels of antibacterial peptide genes such as *Itln1*/*2*, *Retnlb*, and *Ang4* encoding intelectin-1/2, resistin-like molecule-β, and angiogenin-4, respectively (35). As is revealed in Fig 3E, expression of both *Itln1*, known to be involved in bacterial clearance [37] and its homolog *Itln2* [38], decreased significantly in response to CR infection while the levels were further attenuated in the CR+DBZ group. Interestingly, expression of both *Ang4* and *Retnlb* increased following CR infection but declined in the CR+DBZ group (Fig 3E). These results suggest that enteric infections coupled with Notch blockade may be detrimental to the integrity of the colonic mucosa.

### Tight and adherens junctions are disrupted during CR infection and Notch pathway blockade

**Fig 4:**
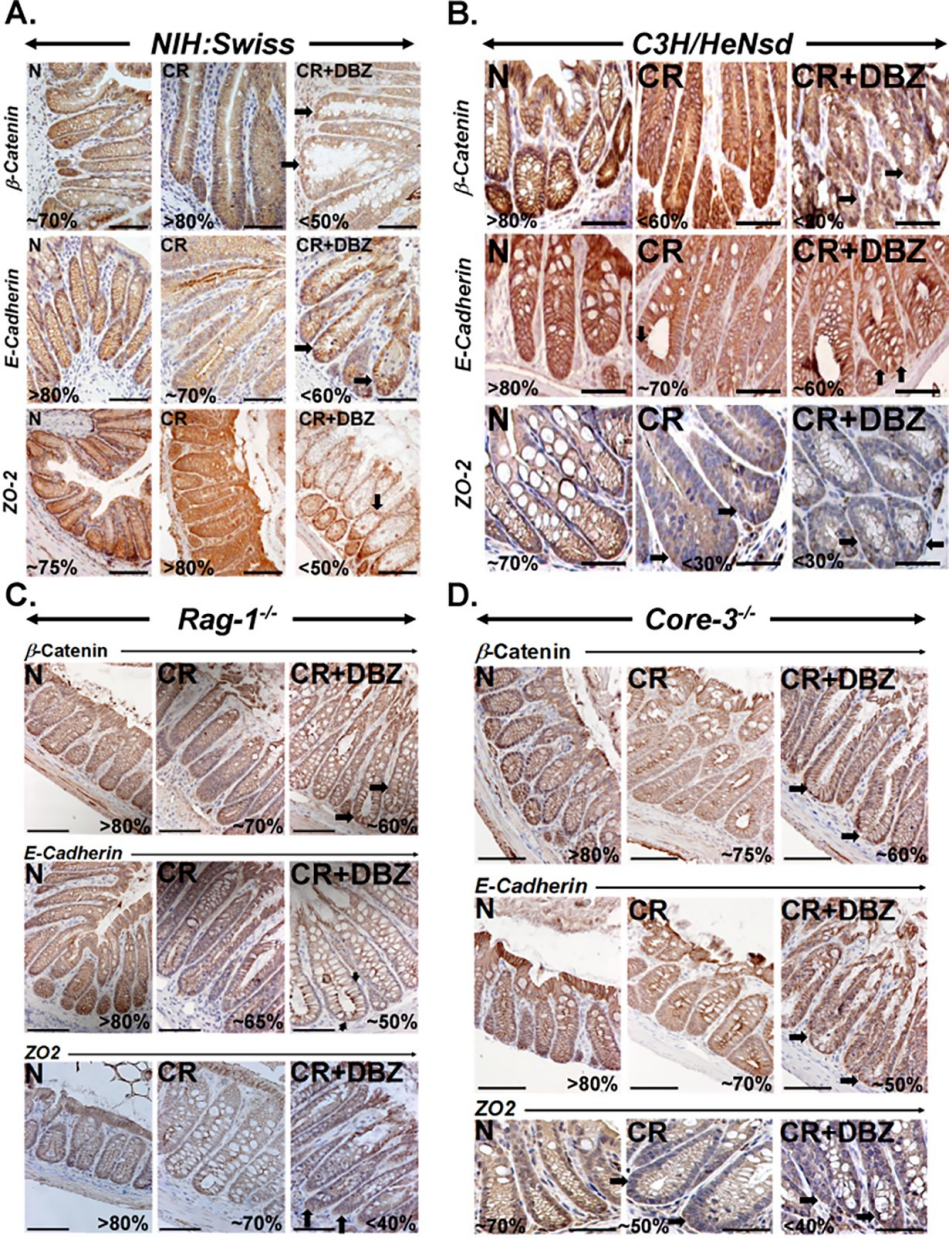
Chronic Notch inhibition and tight and adherens junction integrity. Paraffin-embedded sections prepared from the colons of uninfected (N), CR-infected (CR) and CR+DBZ-treated *NIH*: *Swiss*, *C3H*, *Rag-1*^*-/-*^ and *Core-3*^*-/-*^ mice were stained for β-catenin, E-cadherin and ZO-2, respectively. Arrows highlight the significant loss of these proteins in CR infected or CR+DBZ-treated colons. Bars = 100μm; n = 3 independent experiments.

Since enteric pathogens are implicated in barrier disruption, we hypothesized that CR infection coupled with the Notch pathway blockade may promote alterations in epithelial barrier integrity by modulating proteins involved in the tight and adherens junction (TJ, AJ) formation and maintenance in the colon. We observed a significant loss of TJ protein ZO-2 along with decreases in AJ proteins β-catenin and E-cadherin in the colons of CR+DBZ mice compared to those from untreated or CR-infected mice (Fig 4). Since C3H mice respond more aggressively to CR infection, we also treated CR-infected C3H mice with DBZ and discovered further loss in staining for ZO-2, β-catenin and E-cadherin respectively (Fig 4). We further validated our findings in *Rag-1*^*-/-*^ mice, which are deficient in T and B-cells and lack adaptive immunity. *Rag-1*^*-/-*^ mice have been shown to exhibit transient colitis in response to CR infection [39]. Immunohistochemistry revealed decreased staining for ZO-2, β-catenin, and E-cadherin in the distal colons of CR+DBZ-treated mice (Fig 4). Finally, we utilized *Core-3*^*-/-*^ mice which lack β-1,3-N-acetylglucosaminyltransferase, an enzyme important in the synthesis of core-3-derived O-glycans, the primary component of the intestinal mucin. These mice have defects in the mucus barrier and are susceptible to colitic insult [5]. We have shown previously that *Core-3*^*-/-*^ mice when subjected to CR+DBZ treatment, develop severe inflammation and colitis [16]. In the current study, the staining intensities for ZO-2, β-catenin and E-cadherin proteins declined in CR infected mice and these reductions in protein intensities were exacerbated in CR+DBZ-treated mice (Fig 4). Thus, using mice of varying genetic backgrounds, we clearly demonstrate that loss of junctional proteins corroborate with bacterial invasion and colitis.

### Antibiotic treatment ameliorates colitis and increases survival of CR+DBZ-treated mice

**Fig 5:**
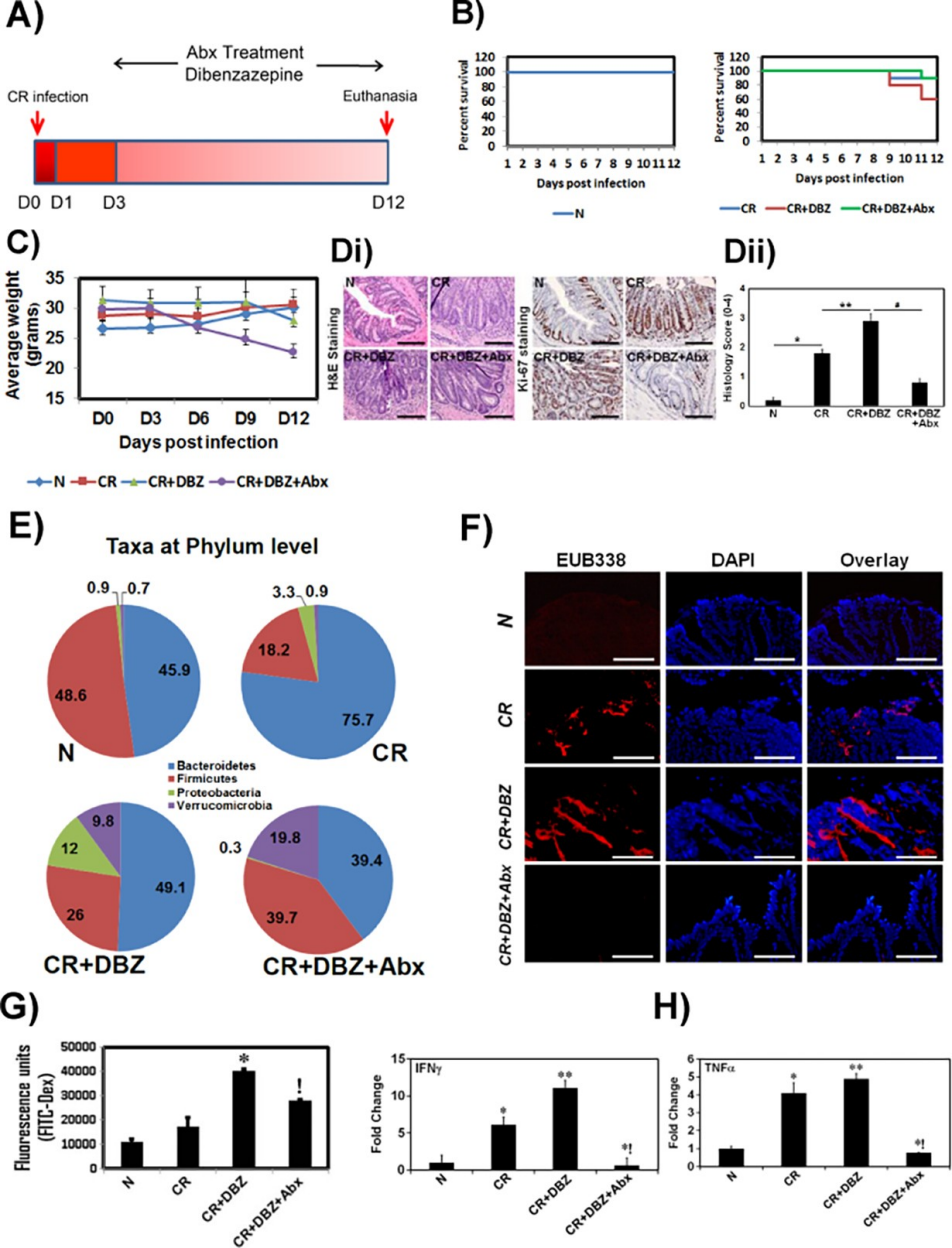
Effect of antibiotics treatment on amelioration of colitis. **A.** Schema of CR infection and DBZ and antibiotics treatment (1g/l metronidazole and 0.2 g/l ciprofloxacin). **B.** Survival kinetics for mice in indicated groups (n = 5 independent experiments). **C.** Periodic weight measurement of mice in various treatment groups (Error bars represent the SD of samples within the group; p< 0.05; n = 5 per group). **D.** Representative hematoxylin and eosin (H&E; left panel) and Ki-67 (right panel) staining of colon sections prepared from the indicated groups (bar = 100μm; n = 5 independent experiments). **E.** The microbial composition at the phyla level in the feces of N, CR, CR+DBZ and CR+DBZ+Abx-treated mice (#s in the chart represent % of each phyla) (n = 10 mice/group). **F.** The bacteria in the colonic tissues of N, CR, CR+DBZ, and CR+DBZ+Abx-treated mice were detected by FISH using a general bacterial 16S probe (red, TexasRed-Eub338; Bar = 100μm; n = 3 independent experiments). DAPI was used as counter-stain. **G.** Mice in indicated groups were subjected to gavage with FITC-D, and serum concentrations, shown as fluorescence units, were measured 4h later. *, p<0.05 versus CR; !, p<0.05 versus CR+DBZ; n = 3 independent experiments. **H.** qPCR to examine expression levels of pro-inflammatory cytokines, IFNγ and TNFα in the indicated groups (*, **p<0.05; n = 3 independent experiments).

**Fig 6:**
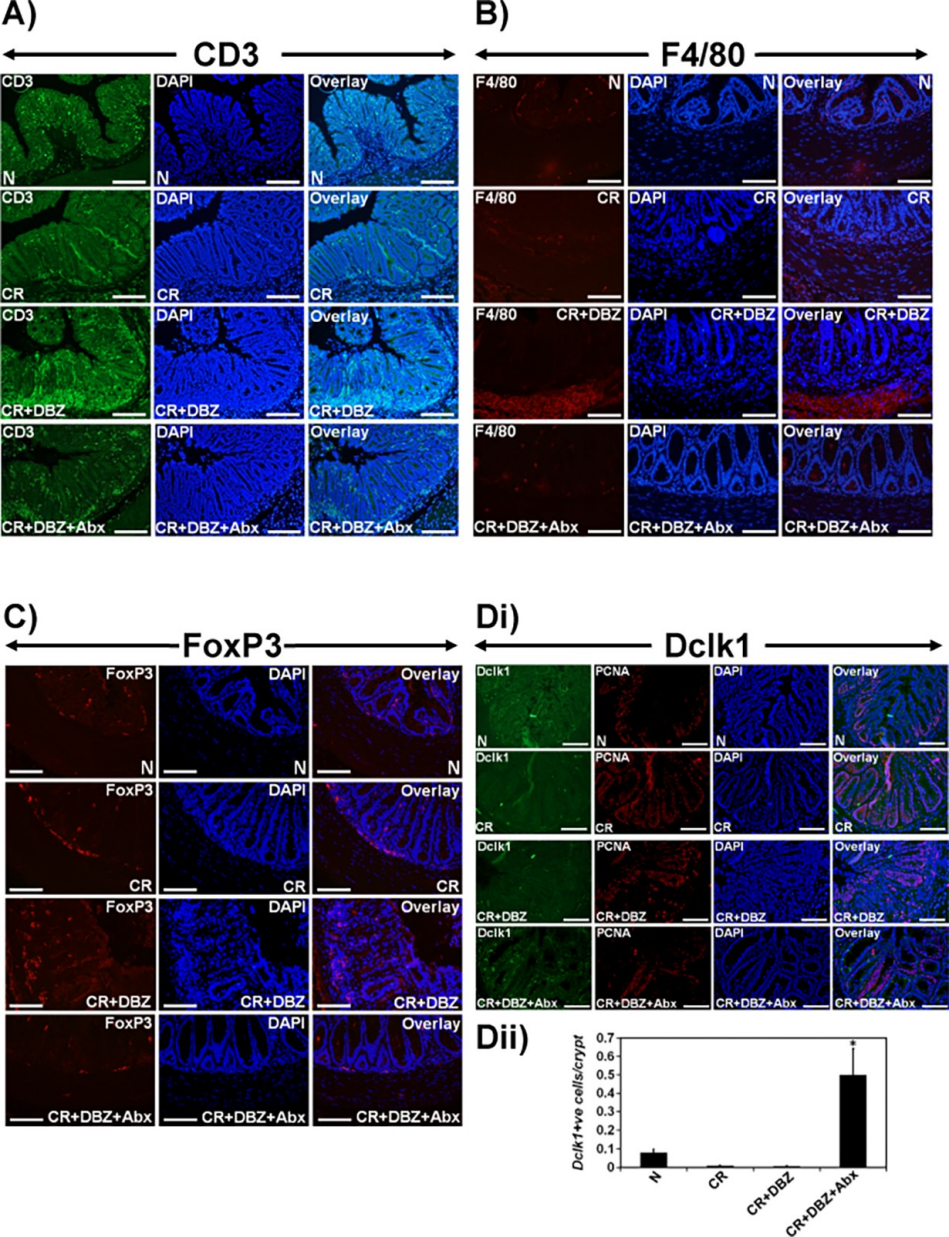
Effect of antibiotics treatment on immune cell recruitment and ISC marker expression. **A-D.** Representative immuno-staining for CD3 (A), F4/80 (B), FoxP3 (C) and Dclk1 (Di) in the colon sections prepared from the indicated groups. Bar = 100μm; n = 3 independent experiments). **Dii.** Quantitative assessment of Dclk1+ cells/crypt; *p<0.05; n = 3 independent experiments.

Since CR+DBZ treatment was associated with significant dysbiosis (Fig 3), we next investigated the hypothesis that antibiotics (Abx) treatment may help ameliorate colitis by blocking increases in potential pathobionts. Fig 5A describes a treatment strategy for NIH: Swiss outbred mice. In response to Abx administration to CR+DBZ mice, the overall survival was in the 60% to 90% range compared to CR+DBZ alone (Fig 5B). Intriguingly, the body weight of CR+DBZ+Abx mice as compared to CR+DBZ mice did not correlate with survival despite the absence of any gross pathology (Fig 5C). Histology of colon sections confirmed a significant reduction in immune cell infiltration following antibiotic treatment (Fig 5D). Since microbiota and microbial metabolites contribute significantly towards epithelial proliferation and turnover, we next examined the impact of microbiota depletion on cellular proliferation. Staining of colon sections with Ki-67, a proliferation marker, revealed that CR+DBZ+Abx mice exhibited even lesser epithelial proliferation than CR+DBZ mice (Fig 5D) suggesting not only that microbiota is required for cellular proliferation but that lack of weight gain in CR+DBZ+Abx group may corroborate with less efficient mucosal recovery. To further examine the effect of antibiotics treatment on 16S rDNA profile, we evaluated fecal samples from various groups. As is revealed in Fig 5E, acute Abx treatment restored *Bacteroidetes* and reduced *Proteobacteria*. Intriguingly, however, the relative abundance of the *Verrucomicrobia* phyla increased in response to antibiotics (Fig 5E). Next, colons from the same group of mice were flushed to remove feces followed by FISH analysis that revealed a reduction in mucosally attached bacteria in the CR+DBZ+Abx group (Fig 5F) that correlated with reduced paracellular permeability (Fig 5G). qPCR of colon tissues from CR+DBZ+Abx group revealed a significant reduction in pro-inflammatory cytokines IFNγ and TNFα compared to either CR or CR+DBZ group (Fig 5H). These results coincided with a significant reduction in CD3+ T cells and F4/80+ macrophages in response to antibiotics compared to CR+DBZ group (Fig6A and 6B). Interestingly, Foxp3+ regulatory T cells that showed elevated levels in CR+DBZ-treated colons did not elicit dramatic changes in the antibiotic-treated group (Fig 6C). This is consistent with Tregs being more common in actively inflamed than uninflamed IBD mucosa [40–43].

Since Notch signaling has been implicated in the maintenance of intestinal stem cells, we next explored the status of putative stem cell marker doublecortin-like kinase 1 (Dclk1) in various treatment groups. In response to CR+DBZ, there was a significant reduction in the levels of Dclk1 (Fig 6Di) consistent with our previous findings [16]. Interestingly, CR+DBZ+Abx group exhibited increased Dclk1 staining that correlated with increases in Dclk1+ cells/crypt (Fig 6Dii). Dclk1 however, did not co-localize with PCNA suggesting that Dclk1+ cells were quiescent cells (Fig 6). These findings indicate a novel role for Dclk1 in promoting maintenance of crypt integrity without necessarily acting as the reservoir for epithelial proliferation.

**Fig 7:**
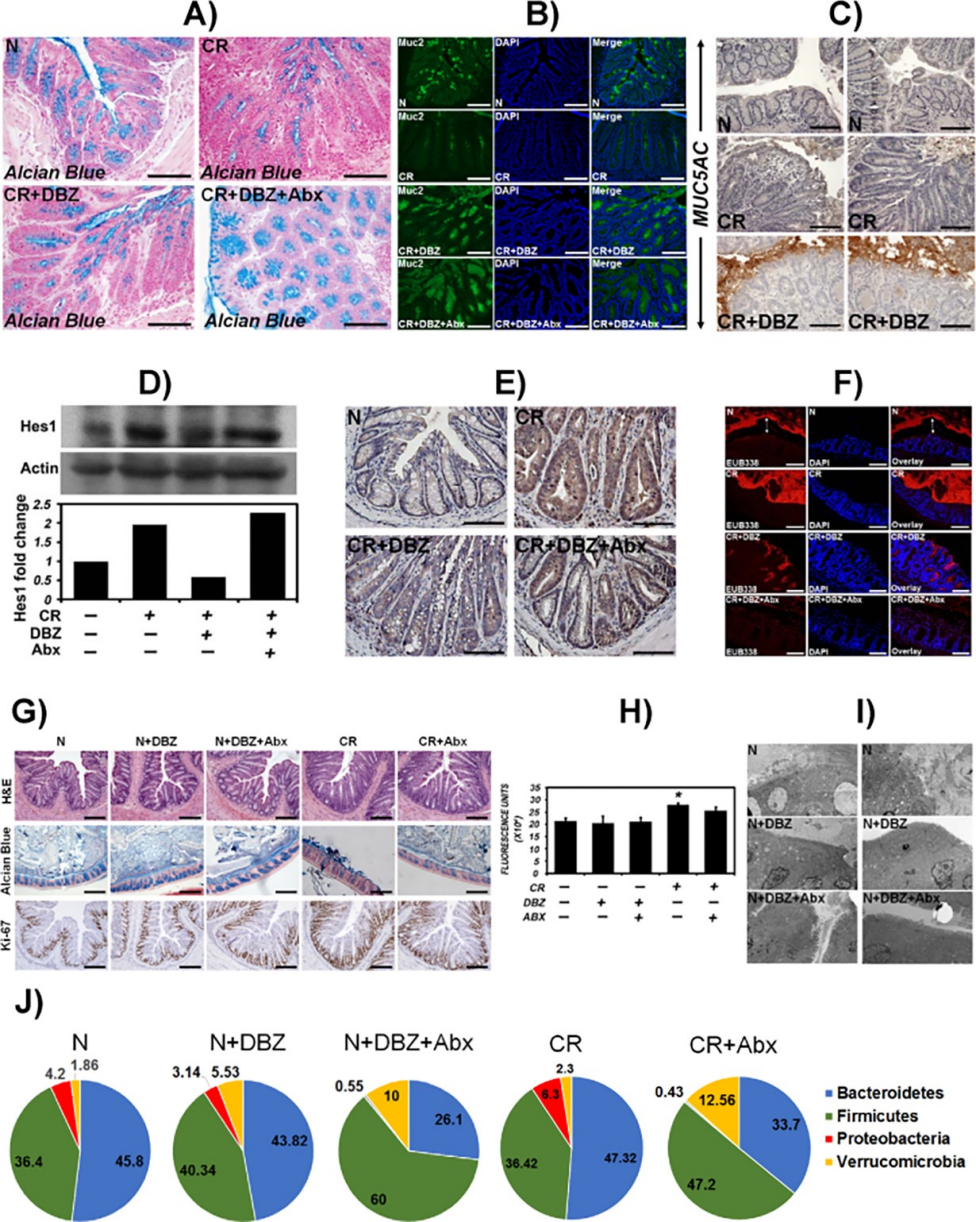
Proof-of-concept experiments to confirm Notch blockade in response to γ-secretase inhibitor, Dibenzazepine (DBZ). NIH: Swiss mice were infected with CR and treated with DBZ, i.p. @ 10μmol/Kg body weight or DBZ+ antibiotics (Abx) (1g/l metronidazole and 0.2 g/l ciprofloxacin) for 10 days. **A, B.** Paraffin sections prepared from uninfected (N), CR-infected (CR), CR+DBZ-treated and CR+DBZ+Abx-treated mice were stained with Alcian blue (A) and Muc-2 (B) to detect goblet cells. **C.** Paraffin sections from uninfected (N), CR-infected (CR) and CR+DBZ-treated mice were stained for MUC5AC. Bars (A-C) = 150-200μm; n = 3 independent experiments. **D.** Western blot showing relative abundance of Hes-1 in crypt extracts prepared from the indicated groups. Bar graph shows fold change in Hes-1 levels (n = 1). **E.** Representative immuno-staining for Hes-1 in the sections prepared from the indicated groups. Bar = 125μm; n = 3 independent experiments. **F.** Representative fluorescence microscopy to detect bacteria in the unflushed and Carnoy-fixed colonic tissues of N, CR, CR+DBZ and CR+DBZ+Abx-treated mice via FISH using a general bacterial 16S probe (TexasRed-Eub338; Bar = 100μm; n = 10 mice/group). DAPI was used as counter-stain. Two-sided arrows represent the distance between the bacterial staining and the epithelial monolayer. **G-J. Control experiments in uninfected (N) mice treated with DBZ or DBZ+Abx and CR-infected mice treated with Abx**. Indicated analyses including H&E, Alcian Blue and Ki-67 staining (G; bar = 75μm), FITC-D measurement as fluorescence units (H; *p<0.05), electron microscopy (I) and 16S sequencing at the phyla level (J) were performed. n = 3 independent experiments.

As a proof-of-concept, we confirmed Notch blockade through pharmacological inhibition via γ-secretase inhibitor DBZ in a series of experiments described in Fig 7. Alcian blue staining confirmed the loss of goblet cells in CR-infected crypts while DBZ treatment of CR-infected mice resulted in the loss of proliferating crypt progenitors due to their conversion into post-mitotic goblet cells. Interestingly, Abx treatment failed to reverse goblet cell hyperplasia (Fig 7A) consistent with its inability to restore epithelial proliferation (Fig 5D). These studies were also consistent with significant Muc-2 staining in both CR+DBZ-treated and CR+DBZ+Abx-treated sections and MUC5AC staining in CR+DBZ-treated sections (Figs 7B and 7C), respectively. Western blotting or immunostaining of Hes-1, a downstream target of Notch signaling, revealed increases in relative abundance in response to CR while DBZ treatment reduced the levels/staining significantly (Fig 7D and 7E) as was shown by us previously [16]. Abx treatment of CR+DBZ mice, however, rescued Hes-1 to some extent (Fig 7 D and 7E). Finally, we fixed unflushed colon tissues in Carnoy’s fixative to capture lumenal and/or mucosally-attached bacteria, particularly in the CR+DBZ group, via EUB338 staining (Fig 7F). Upon Abx treatment, crypt invasion was significantly attenuated (Fig 7F). We have shown previously that DBZ alone in the absence of CR infection, had no toxicity or adverse effects in the gut [16]. As is depicted in Fig 7G–7J, data on histology, cell proliferation, electron microscopy, microbiome, and mucosal permeability further clarify that treatment of control mice with DBZ has no effects on any of these parameters and that DBZ on its own, is not toxic. These results establish the reproducibility of our experimental strategies.

## Discussion

This study delineated the role of enteric infection and Notch pathway blockade in microbial dysbiosis, severe inflammation, and colitis-like disease development in outbred mice that typically recover from a self-limiting disease [18]. We report alterations in mucus composition that coincides with microbial dysbiosis, bacterial dissemination to the epithelial monolayers and disruption of epithelial junctions preceding and/or accompanying immune cell activation and colitis-like disease development.

The human colon is a reservoir for mucus whose secretion is largely controlled by multiple factors including LPS and lipoteichoic acid, cytokines, and hormones produced during inflammation [36]. Changes in mucin glycosylation alter the supply of carbohydrates available to bacteria utilizing mucin glycans as a carbon source, thereby changing the microbiota composition. We have observed altered *N*- and *O*-glycosylation pattern with a significant loss in fucosylated *N*-glycans in CR+DBZ group which is consistent with previous report wherein, the repression of LEE virulence genes by enterohaemorrhagic *E*. *coli* was mediated by increased concentration of free fucose in the colon [44]. The significance of mucin glycans is further highlighted in several mouse models deficient in glycosyltransferases. Transgenic mice lacking Core-1β1,3-galactosyltransferase (C1GalT1, also called T-synthase) and Core 3β1,3 *N*-acetylglucosaminyltransferase (C3Gnt) are highly susceptible to inflammatory insults including dextran sodium sulfate (DSS) challenge [5, 6, 45]. The chemical diversity of endogenous *O*- and *N*-linked glycans requires that mucosal bacteria produce many different degradative enzymes to effectively use these heterogeneous polymers. While *O*-glycans most likely impart structural stability to the mucus layers of the distal colon, they are increasingly appreciated as important in shaping the composition of the microbial communities within the gut [46]. For *Escherichia coli*, the *O*-glycans present in secreted mucins may also impact the ability of some bacteria to thrive after they colonize [47], suggesting a nutritional basis for selection. The ability to extract carbohydrates from mucin glycans is concentrated in bacterial groups, which express a vast set of hydrolase enzymes and transporters enabling the utilization of monosaccharides as carbon sources. Among the bacterial phyla of the human gut, *Bacteroidetes* express the largest carbohydrate-fermenting machinery. Several *Firmicutes*, such as *Ruminococcus intestinalis*, *R*. *gnavus* and *R*. *flavefaciens* also express >100 carbohydrate degrading enzymes per genome and are capable of digesting mucin glycans [48]. Members of *Proteobacteria*, such as *Enterobacteriaceae* in contrast, have limited ability to degrade intestinal mucins. Among *Actinobacteria*, several *Bifidobacterium* spp. are specialized at fermenting complex fucosylated oligosaccharides [49] while *A*. *muciniphila*, a member of the *Verrucomicrobiota* phylum, is another microbe specialized in the utilization of intestinal mucins as carbon source [50]. We discovered significant loss in *Bacteroidetes* while *Proteobacteria* and *Verrucomicrobiota* phyla showed prominent increases in the CR+DBZ group. In particular, we saw *A*. *mucinophila* to increase significantly and coincide with changes in mucus composition and microbial dysbiosis. Earle et al., using imaging methods, recently showed a bloom of the *Akkermansia* population in mice following a depletion of microbiota accessible carbohydrates, resulting in a thinner mucus layer in the distal colon [51]. Another study similarly observed *Akkermansia* to increase markedly after DSS treatment [52], while this phenomenon was not always reproducible in other studies [53]. One could speculate that the thickness of the mucus layer and the observed low-grade inflammation in the DSS mice may negatively influence *A*. *muciniphila* colonization. Similarly, Ganesh BP et al. [54] also observed increased *A*. *muciniphila* during *Salmonella Typhimurium* infection. It is interesting that *A*. *muciniphila* is a commensal bacterium in the mammalian gastrointestinal tract belonging to family *Verrucomicrobiaceae* that adheres to the mucus layer and has an important role in maintaining gut barrier function [50, 55, 56]. Support for the beneficial effect of *Akkermansia* on colitis comes from the observation that extracellular vesicles from *A*. *muciniphila* were found to protect against the DSS-induced phenotype [53]. In most human studies, a depletion of *A*. *muciniphila* is observed in IBD mucosa and in fecal samples from ulcerative colitis patients [57, 58]. It is therefore probable that altered lumenal/mucosal microenvironment may convert *A*. *muciniphila* into a potential pathobiont that further influences inflammatory signaling. Regardless of whether it promotes barrier disruption and colitis or maintains physiological mucus production in the gut, the impact of mucin carbohydrate on microbiota changes merits further investigation.

Although tight junctions efficiently restrict most microbes from penetrating into deeper tissues and contain the microbiota, some pathogens have developed specific strategies to alter or disrupt these structures as part of their pathogenesis, resulting in either pathogen penetration or consequences such as diarrhea. Since delineating the strategies that microorganisms use to regulate the functions of tight junctions is paramount to understanding the role of microbiota in disease pathogenesis, we analyzed the contribution of Notch signaling in the maintenance of tight and adherens junction proteins in our model. In this report, we show that during *C*. *rodentium* infection and in the absence of Notch signaling, there is impairment of TJ and AJ proteins which leads to increased permeability and hence the exposure of luminal contents to immune system eliciting inflammation [59]. Under physiological conditions, the gastrointestinal epithelium expresses TJ proteins that prevent luminal antigens from penetrating into deeper tissues. However, various pathological insults, including enteric pathogens, may compromise this function. For example, TJs are altered during infection with *C*. *rodentium* resulting in a functionally deficient epithelial cell barrier [60]. *Giardia lamblia* [61], *Helicobacter pylori* [62], and rotavirus [63] have all evolved the ability to disrupt intestinal epithelial TJs, leading to increased paracellular permeability. Similarly, extensive *in vitro* studies have determined that *EPEC* is capable of altering TJ integrity and paracellular permeability in cultured human IECs [64]. In IBD, a dysfunction of AJ proteins has been described and consists of downregulation of E-Cadherin, which weakens intercellular adhesion and promotes inflammatory response [65]. Our studies further add to this discussion by providing insight into the role of enteric infection in facilitating intestinal barrier disruption and ensuing inflammation leading to the development of infectious colitis.

Alterations in gut microbiota and specifically reduced intestinal microbial diversity, have been found to be associated with chronic gut inflammation in inflammatory bowel disease (IBD). Specific bacterial pathogens, such as virulent *Escherichia coli* strains, *Bacteroides* spp, and *Mycobacterium avium* subspecies *paratuberculosis*, have been linked to the pathogenesis of IBD. Antibiotics may influence the course of these diseases by decreasing concentrations of bacteria in the gut lumen and altering the composition of intestinal microbiota. Different antibiotics, including ciprofloxacin, metronidazole, the combination of both, rifaximin, and anti-tuberculous regimens have been evaluated in clinical trials for the treatment of IBD [66]. A combination of metronidazole and ciprofloxacin decreased the intensity of the bacterial attachment to the colonic mucosa and attenuated inflammation in CR+DBZ+Abx group (Fig 5). Intriguingly, however, the antibiotic cocktail failed to diminish or eliminate the *Verrucomicrobia* phyla that exhibited an almost 2-fold increase in relative abundance following Abx treatment. Since antibiotics reduce/eliminate key commensal bacteria that provide colonization resistance [67], it is tempting to speculate that this may have lowered the threshold required for *Verrucomicrobia* phyla to flourish resulting in a 2-fold increase. These studies underscore the complexity of gut microbiota and suggest a complex regulatory mechanism worthy of future investigations. In general, however, the protective role of Abx was evident since there were increased survival rates, decreased proinflammatory cytokines and immune cells in the colonic mucosa. The protective role of metronidazole or the combination of metronidazole and ciprofloxacin in acute DSS colitis model has been shown by several other studies [68, 69].

Previous studies have investigated therapy based on stem cell administration for the treatment of IBD-like diseases in mice. Amelioration of experimental colitis has been described using hematopoietic stem cells [70], mesenchymal stem cells [71] and colonic stem cells [72]. In response to Abx treatment, we observed a significant increase in Dclk1+ cells that correlated with increased animal survival and amelioration of colitis-like symptoms. How increases in endogenous Dclk1 contribute towards disease amelioration and survival remains to be determined and is currently being investigated in our laboratory.

In conclusion, we report a novel mechanism wherein, enteric infection coupled with chronic Notch pathway inhibition is associated with changes in mucus composition, bacterial dysbiosis and loss of tight junction integrity that leads to severe inflammation and colitis. Our findings also suggest that controlled use of antibiotics may alleviate gut dysbiosis but may not be sufficient to promote complete mucosal recovery.

## References

[1] Birchenough GM, Nystrom EE, Johansson ME, Hansson GC. A sentinel goblet cell guards the colonic crypt by triggering Nlrp6-dependent Muc2 secretion. Science. 2016;352(6293):1535–42. Epub 2016/06/25. 10.1126/science.aaf7419 . 27339979 PMC5148821

[2] McGuckin MA, Linden SK, Sutton P, Florin TH. Mucin dynamics and enteric pathogens. Nature reviews Microbiology. 2011;9(4):265–78. Epub 2011/03/17. 10.1038/nrmicro2538 . 21407243

[3] Johansson ME, Phillipson M, Petersson J, Velcich A, Holm L, Hansson GC. The inner of the two Muc2 mucin-dependent mucus layers in colon is devoid of bacteria. Proc Natl Acad Sci U S A. 2008;105(39):15064–9. Epub 2008/09/23. 10.1073/pnas.0803124105 ; PubMed Central PMCID: PMC2567493. 18806221 PMC2567493

[4] Hansson GC. Role of mucus layers in gut infection and inflammation. Curr Opin Microbiol. 2011 Epub 2011/12/20. 10.1016/j.mib.2011.11.002 . 22177113 PMC3716454

[5] An G, Wei B, Xia B, McDaniel JM, Ju T, Cummings RD, et al Increased susceptibility to colitis and colorectal tumors in mice lacking core 3-derived O-glycans. J Exp Med. 2007;204(6):1417–29. Epub 2007/05/23. 10.1084/jem.20061929 ; PubMed Central PMCID: PMCPMC2118614. 17517967 PMC2118614

[6] Fu J, Wei B, Wen T, Johansson ME, Liu X, Bradford E, et al Loss of intestinal core 1-derived O-glycans causes spontaneous colitis in mice. J Clin Invest. 2011;121(4):1657–66. Epub 2011/03/09. 10.1172/JCI45538 ; PubMed Central PMCID: PMCPMC3069788. 21383503 PMC3069788

[7] Johansson ME, Larsson JM, Hansson GC. The two mucus layers of colon are organized by the MUC2 mucin, whereas the outer layer is a legislator of host-microbial interactions. Proc Natl Acad Sci U S A. 2011;108 Suppl 1:4659–65. Epub 2010/07/10. 10.1073/pnas.1006451107 ; PubMed Central PMCID: PMCPmc3063600. 20615996 PMC3063600

[8] Backhed F, Ley RE, Sonnenburg JL, Peterson DA, Gordon JI. Host-bacterial mutualism in the human intestine. Science. 2005;307(5717):1915–20. Epub 2005/03/26. 10.1126/science.1104816 . 15790844

[9] Backhed F. Host responses to the human microbiome. Nutr Rev. 2012;70 Suppl 1:S14–7. Epub 2012/08/17. 10.1111/j.1753-4887.2012.00496.x . 22861802

[10] Tremaroli V, Kovatcheva-Datchary P, Backhed F. A role for the gut microbiota in energy harvesting? Gut. 2010;59(12):1589–90. Epub 2010/10/14. 10.1136/gut.2010.223594 . 20940281

[11] Scaldaferri F, Pizzoferrato M, Gerardi V, Lopetuso L, Gasbarrini A. The gut barrier: new acquisitions and therapeutic approaches. J Clin Gastroenterol. 2012;46 Suppl:S12–7. Epub 2012/09/14. 10.1097/MCG.0b013e31826ae849 . 22955350

[12] Xavier RJ, Podolsky DK. Unravelling the pathogenesis of inflammatory bowel disease. Nature. 2007;448(7152):427–34. Epub 2007/07/27. 10.1038/nature06005 . 17653185

[13] Dahan S, Rabinowitz KM, Martin AP, Berin MC, Unkeless JC, Mayer L. Notch-1 signaling regulates intestinal epithelial barrier function, through interaction with CD4+ T cells, in mice and humans. Gastroenterology. 2011;140(2):550–9. Epub 2010/11/09. 10.1053/j.gastro.2010.10.057 ; PubMed Central PMCID: PMCPmc3031772. 21056041 PMC3031772

[14] Pope JL, Bhat AA, Sharma A, Ahmad R, Krishnan M, Washington MK, et al Claudin-1 regulates intestinal epithelial homeostasis through the modulation of Notch-signalling. Gut. 2014;63(4):622–34. Epub 2013/06/15. 10.1136/gutjnl-2012-304241 ; PubMed Central PMCID: PMCPmc4083824. 23766441 PMC4083824

[15] van Es JH, van Gijn ME, Riccio O, van den Born M, Vooijs M, Begthel H, et al Notch/gamma-secretase inhibition turns proliferative cells in intestinal crypts and adenomas into goblet cells. Nature. 2005;435(7044):959–63. Epub 2005/06/17. 10.1038/nature03659 . 15959515

[16] Ahmed I, Chandrakesan P, Tawfik O, Xia L, Anant S, Umar S. Critical Roles of Notch and Wnt/beta-Catenin Pathways in the Regulation of Hyperplasia and/or Colitis in Response to Bacterial Infection. Infect Immun. 2012;80(9):3107–21. Epub 2012/06/20. 10.1128/IAI.00236-12 ; PubMed Central PMCID: PMCPMC3418747. 22710872 PMC3418747

[17] Chandrakesan P, Ahmed I, Chinthalapally A, Singh P, Awasthi S, Anant S, et al Distinct Compartmentalization of NF-kappaB Activity in Crypt and Crypt-Denuded Lamina Propria Precedes and Accompanies Hyperplasia and/or Colitis following Bacterial Infection. Infect Immun. 2012;80(2):753–67. Epub 2011/12/07. 10.1128/IAI.06101-11 ; PubMed Central PMCID: PMC3264290. 22144489 PMC3264290

[18] Sellin JH, Wang Y, Singh P, Umar S. beta-Catenin stabilization imparts crypt progenitor phenotype to hyperproliferating colonic epithelia. Exp Cell Res. 2009;315(1):97–109. Epub 2008/11/11. 10.1016/j.yexcr.2008.10.019 ; PubMed Central PMCID: PMC2868370. 18996369 PMC2868370

[19] Sellin JH, Umar S, Xiao J, Morris AP. Increased beta-catenin expression and nuclear translocation accompany cellular hyperproliferation in vivo. Cancer Res. 2001;61(7):2899–906. Epub 2001/04/18. . 11306465

[20] Wang Y, Xiang GS, Kourouma F, Umar S. Citrobacter rodentium-induced NF-kappaB activation in hyperproliferating colonic epithelia: role of p65 (Ser536) phosphorylation. Br J Pharmacol. 2006;148(6):814–24. Epub 2006/06/06. 10.1038/sj.bjp.0706784 ; PubMed Central PMCID: PMC1617077. 16751795 PMC1617077

[21] Chandrakesan P, Roy B, Jakkula LU, Ahmed I, Ramamoorthy P, Tawfik O, et al Utility of a bacterial infection model to study epithelial-mesenchymal transition, mesenchymal-epithelial transition or tumorigenesis. Oncogene. 2013 Epub 2013/06/12. 10.1038/onc.2013.210 . 23752178 PMC3883801

[22] Chandrakesan P, Ahmed I, Anwar T, Wang Y, Sarkar S, Singh P, et al Novel changes in NF-{kappa}B activity during progression and regression phases of hyperplasia: role of MEK, ERK, and p38. J Biol Chem. 2010;285(43):33485–98. Epub 2010/08/17. 10.1074/jbc.M110.129353 ; PubMed Central PMCID: PMC2963366. 20710027 PMC2963366

[23] Umar S, Morris AP, Kourouma F, Sellin JH. Dietary pectin and calcium inhibit colonic proliferation in vivo by differing mechanisms. Cell Prolif. 2003;36(6):361–75. Epub 2004/01/09. . 14710853 10.1046/j.1365-2184.2003.00291.x PMC6496283

[24] Rengifo-Cam W, Umar S, Sarkar S, Singh P. Antiapoptotic effects of progastrin on pancreatic cancer cells are mediated by sustained activation of nuclear factor-{kappa}B. Cancer Res. 2007;67(15):7266–74. Epub 2007/08/03. 10.1158/0008-5472.CAN-07-1206 . 17671195

[25] Ahmed I, Roy B, Chandrakesan P, Venugopal A, Xia L, Jensen R, et al Evidence of functional cross talk between the Notch and NF-kappaB pathways in nonneoplastic hyperproliferating colonic epithelium. Am J Physiol Gastrointest Liver Physiol. 2013;304(4):G356–70. Epub 2012/12/04. 10.1152/ajpgi.00372.2012 ; PubMed Central PMCID: PMCPMC3566617. 23203159 PMC3566617

[26] Barthold SW, Osbaldiston GW, Jonas AM. Dietary, bacterial, and host genetic interactions in the pathogenesis of transmissible murine colonic hyperplasia. Lab Anim Sci. 1977;27(6):938–45. Epub 1977/12/01. . 599885

[27] Milano J, McKay J, Dagenais C, Foster-Brown L, Pognan F, Gadient R, et al Modulation of notch processing by gamma-secretase inhibitors causes intestinal goblet cell metaplasia and induction of genes known to specify gut secretory lineage differentiation. Toxicol Sci. 2004;82(1):341–58. Epub 2004/08/21. 10.1093/toxsci/kfh254 . 15319485

[28] Roy BC, Subramaniam D, Ahmed I, Jala VR, Hester CM, Greiner KA, et al Role of bacterial infection in the epigenetic regulation of Wnt antagonist WIF1 by PRC2 protein EZH2. Oncogene. 2014 Epub 2014/12/09. 10.1038/onc.2014.386 . 25486432 PMC4459936

[29] Ahmed I, Roy BC, Subramaniam D, Ganie SA, Kwatra D, Dixon D, et al An ornamental plant targets epigenetic signaling to block cancer stem cell-driven colon carcinogenesis. Carcinogenesis. 2016;37(4):385–96. Epub 2016/01/21. 10.1093/carcin/bgw009 ; PubMed Central PMCID: PMCPmc4806710. 26785732 PMC4806710

[30] Caporaso JG, Lauber CL, Walters WA, Berg-Lyons D, Huntley J, Fierer N, et al Ultra-high-throughput microbial community analysis on the Illumina HiSeq and MiSeq platforms. The ISME journal. 2012;6(8):1621–4. Epub 2012/03/10. 10.1038/ismej.2012.8 ; PubMed Central PMCID: PMCPmc3400413. 22402401 PMC3400413

[31] Caporaso JG, Kuczynski J, Stombaugh J, Bittinger K, Bushman FD, Costello EK, et al QIIME allows analysis of high-throughput community sequencing data. Nature methods. 2010;7(5):335–6. Epub 2010/04/13. 10.1038/nmeth.f.303 ; PubMed Central PMCID: PMCPmc3156573. 20383131 PMC3156573

[32] Edgar RC. Search and clustering orders of magnitude faster than BLAST. Bioinformatics. 2010;26(19):2460–1. Epub 2010/08/17. 10.1093/bioinformatics/btq461 . 20709691

[33] Price MN, Dehal PS, Arkin AP. FastTree 2—approximately maximum-likelihood trees for large alignments. PLoS One. 2010;5(3):e9490 Epub 2010/03/13. 10.1371/journal.pone.0009490 ; PubMed Central PMCID: PMCPmc2835736. 20224823 PMC2835736

[34] Napolitano LM, Koruda MJ, Meyer AA, Baker CC. The impact of femur fracture with associated soft tissue injury on immune function and intestinal permeability. Shock. 1996;5(3):202–7. Epub 1996/03/01. . 8696984 10.1097/00024382-199603000-00006

[35] Shajahan A, Heiss C, Ishihara M, Azadi P. Glycomic and glycoproteomic analysis of glycoproteins-a tutorial. Anal Bioanal Chem. 2017;409(19):4483–505. Epub 2017/06/07. 10.1007/s00216-017-0406-7 ; PubMed Central PMCID: PMCPmc5498624. 28585084 PMC5498624

[36] Kim YS, Ho SB. Intestinal goblet cells and mucins in health and disease: recent insights and progress. Current gastroenterology reports. 2010;12(5):319–30. Epub 2010/08/13. 10.1007/s11894-010-0131-2 ; PubMed Central PMCID: PMCPmc2933006. 20703838 PMC2933006

[37] Tsuji S, Yamashita M, Hoffman DR, Nishiyama A, Shinohara T, Ohtsu T, et al Capture of heat-killed Mycobacterium bovis bacillus Calmette-Guerin by intelectin-1 deposited on cell surfaces. Glycobiology. 2009;19(5):518–26. Epub 2009/01/31. 10.1093/glycob/cwp013 ; PubMed Central PMCID: PMCPmc2667160. 19179460 PMC2667160

[38] Pemberton AD, Knight PA, Gamble J, Colledge WH, Lee JK, Pierce M, et al Innate BALB/c enteric epithelial responses to Trichinella spiralis: inducible expression of a novel goblet cell lectin, intelectin-2, and its natural deletion in C57BL/10 mice. J Immunol. 2004;173(3):1894–901. Epub 2004/07/22. . 15265922 10.4049/jimmunol.173.3.1894

[39] Vallance BA, Deng W, Knodler LA, Finlay BB. Mice lacking T and B lymphocytes develop transient colitis and crypt hyperplasia yet suffer impaired bacterial clearance during Citrobacter rodentium infection. Infect Immun. 2002;70(4):2070–81. Epub 2002/03/16. 10.1128/IAI.70.4.2070-2081.2002 ; PubMed Central PMCID: PMCPMC127821. 11895973 PMC127821

[40] Maul J, Loddenkemper C, Mundt P, Berg E, Giese T, Stallmach A, et al Peripheral and intestinal regulatory CD4+ CD25(high) T cells in inflammatory bowel disease. Gastroenterology. 2005;128(7):1868–78. Epub 2005/06/09. . 15940622 10.1053/j.gastro.2005.03.043

[41] Lord JD, Valliant-Saunders K, Hahn H, Thirlby RC, Ziegler SF. Paradoxically increased FOXP3+ T cells in IBD do not preferentially express the isoform of FOXP3 lacking exon 2. Dig Dis Sci. 2012;57(11):2846–55. Epub 2012/06/28. 10.1007/s10620-012-2292-3 ; PubMed Central PMCID: PMCPmc3482978. 22736020 PMC3482978

[42] Saruta M, Yu QT, Fleshner PR, Mantel PY, Schmidt-Weber CB, Banham AH, et al Characterization of FOXP3+CD4+ regulatory T cells in Crohn's disease. Clin Immunol. 2007;125(3):281–90. Epub 2007/09/28. 10.1016/j.clim.2007.08.003 . 17897887

[43] Yu QT, Saruta M, Avanesyan A, Fleshner PR, Banham AH, Papadakis KA. Expression and functional characterization of FOXP3+ CD4+ regulatory T cells in ulcerative colitis. Inflamm Bowel Dis. 2007;13(2):191–9. Epub 2007/01/09. 10.1002/ibd.20053 . 17206665

[44] Pacheco AR, Curtis MM, Ritchie JM, Munera D, Waldor MK, Moreira CG, et al Fucose sensing regulates bacterial intestinal colonization. Nature. 2012;492(7427):113–7. Epub 2012/11/20. 10.1038/nature11623 ; PubMed Central PMCID: PMCPmc3518558. 23160491 PMC3518558

[45] Thomsson KA, Holmen-Larsson JM, Angstrom J, Johansson ME, Xia L, Hansson GC. Detailed O-glycomics of the Muc2 mucin from colon of wild-type, core 1- and core 3-transferase-deficient mice highlights differences compared with human MUC2. Glycobiology. 2012;22(8):1128–39. Epub 2012/05/15. 10.1093/glycob/cws083 ; PubMed Central PMCID: PMCPMC3382349. 22581805 PMC3382349

[46] Staubach F, Kunzel S, Baines AC, Yee A, McGee BM, Backhed F, et al Expression of the blood-group-related glycosyltransferase B4galnt2 influences the intestinal microbiota in mice. The ISME journal. 2012;6(7):1345–55. Epub 2012/01/27. 10.1038/ismej.2011.204 ; PubMed Central PMCID: PMCPMC3379640. 22278669 PMC3379640

[47] Chang D-E, Smalley DJ, Tucker DL, Leatham MP, Norris WE, Stevenson SJ, et al Carbon nutrition of Escherichia coli in the mouse intestine. Proc Natl Acad Sci U S A. 2004;101(19):7427–32. 10.1073/pnas.0307888101 15123798 PMC409935

[48] Cockburn DW, Koropatkin NM. Polysaccharide Degradation by the Intestinal Microbiota and Its Influence on Human Health and Disease. J Mol Biol. 2016;428(16):3230–52. Epub 2016/07/10. 10.1016/j.jmb.2016.06.021 . 27393306

[49] Chassard C, Lacroix C. Carbohydrates and the human gut microbiota. Curr Opin Clin Nutr Metab Care. 2013;16(4):453–60. Epub 2013/05/31. 10.1097/MCO.0b013e3283619e63 . 23719143

[50] Derrien M, Collado MC, Ben-Amor K, Salminen S, de Vos WM. The Mucin degrader Akkermansia muciniphila is an abundant resident of the human intestinal tract. Appl Environ Microbiol. 2008;74(5):1646–8. Epub 2007/12/18. 10.1128/AEM.01226-07 ; PubMed Central PMCID: PMCPmc2258631. 18083887 PMC2258631

[51] Earle KA, Billings G, Sigal M, Lichtman JS, Hansson GC, Elias JE, et al Quantitative Imaging of Gut Microbiota Spatial Organization. Cell host & microbe. 2015;18(4):478–88. Epub 2015/10/07. 10.1016/j.chom.2015.09.002 ; PubMed Central PMCID: PMCPmc4628835. 26439864 PMC4628835

[52] Hakansson A, Tormo-Badia N, Baridi A, Xu J, Molin G, Hagslatt ML, et al Immunological alteration and changes of gut microbiota after dextran sulfate sodium (DSS) administration in mice. Clin Exp Med. 2015;15(1):107–20. Epub 2014/01/15. 10.1007/s10238-013-0270-5 ; PubMed Central PMCID: PMCPmc4308640. 24414342 PMC4308640

[53] Kang CS, Ban M, Choi EJ, Moon HG, Jeon JS, Kim DK, et al Extracellular vesicles derived from gut microbiota, especially Akkermansia muciniphila, protect the progression of dextran sulfate sodium-induced colitis. PLoS One. 2013;8(10):e76520 Epub 2013/11/10. 10.1371/journal.pone.0076520 ; PubMed Central PMCID: PMCPmc3811976. 24204633 PMC3811976

[54] Ganesh BP, Klopfleisch R, Loh G, Blaut M. Commensal Akkermansia muciniphila exacerbates gut inflammation in Salmonella Typhimurium-infected gnotobiotic mice. PLoS One. 2013;8(9):e74963 Epub 2013/09/17. 10.1371/journal.pone.0074963 ; PubMed Central PMCID: PMCPmc3769299. 24040367 PMC3769299

[55] Everard A, Belzer C, Geurts L, Ouwerkerk JP, Druart C, Bindels LB, et al Cross-talk between Akkermansia muciniphila and intestinal epithelium controls diet-induced obesity. Proc Natl Acad Sci U S A. 2013;110(22):9066–71. Epub 2013/05/15. 10.1073/pnas.1219451110 ; PubMed Central PMCID: PMCPmc3670398. 23671105 PMC3670398

[56] Derrien M, Van Baarlen P, Hooiveld G, Norin E, Muller M, de Vos WM. Modulation of Mucosal Immune Response, Tolerance, and Proliferation in Mice Colonized by the Mucin-Degrader Akkermansia muciniphila. Frontiers in microbiology. 2011;2:166 Epub 2011/09/10. 10.3389/fmicb.2011.00166 ; PubMed Central PMCID: PMCPmc3153965. 21904534 PMC3153965

[57] Png CW, Linden SK, Gilshenan KS, Zoetendal EG, McSweeney CS, Sly LI, et al Mucolytic bacteria with increased prevalence in IBD mucosa augment in vitro utilization of mucin by other bacteria. Am J Gastroenterol. 2010;105(11):2420–8. Epub 2010/07/22. 10.1038/ajg.2010.281 . 20648002

[58] Rajilic-Stojanovic M, Shanahan F, Guarner F, de Vos WM. Phylogenetic analysis of dysbiosis in ulcerative colitis during remission. Inflamm Bowel Dis. 2013;19(3):481–8. Epub 2013/02/07. 10.1097/MIB.0b013e31827fec6d . 23385241

[59] Grivennikov SI, Wang K, Mucida D, Stewart CA, Schnabl B, Jauch D, et al Adenoma-linked barrier defects and microbial products drive IL-23/IL-17-mediated tumour growth. Nature. 2012;491(7423):254–8. Epub 2012/10/05. 10.1038/nature11465 ; PubMed Central PMCID: PMCPmc3601659. 23034650 PMC3601659

[60] Guttman JA, Samji FN, Li Y, Vogl AW, Finlay BB. Evidence that tight junctions are disrupted due to intimate bacterial contact and not inflammation during attaching and effacing pathogen infection in vivo. Infect Immun. 2006;74(11):6075–84. Epub 2006/09/07. 10.1128/IAI.00721-06 ; PubMed Central PMCID: PMCPMC1695516. 16954399 PMC1695516

[61] Chin AC, Teoh DA, Scott KG, Meddings JB, Macnaughton WK, Buret AG. Strain-dependent induction of enterocyte apoptosis by Giardia lamblia disrupts epithelial barrier function in a caspase-3-dependent manner. Infect Immun. 2002;70(7):3673–80. Epub 2002/06/18. 10.1128/IAI.70.7.3673-3680.2002 ; PubMed Central PMCID: PMCPMC128105. 12065509 PMC128105

[62] Fedwick JP, Lapointe TK, Meddings JB, Sherman PM, Buret AG. Helicobacter pylori activates myosin light-chain kinase to disrupt claudin-4 and claudin-5 and increase epithelial permeability. Infect Immun. 2005;73(12):7844–52. Epub 2005/11/22. 10.1128/IAI.73.12.7844-7852.2005 ; PubMed Central PMCID: PMCPMC1307049. 16299274 PMC1307049

[63] Dickman KG, Hempson SJ, Anderson J, Lippe S, Zhao L, Burakoff R, et al Rotavirus alters paracellular permeability and energy metabolism in Caco-2 cells. Am J Physiol Gastrointest Liver Physiol. 2000;279(4):G757–66. Epub 2000/09/27. 10.1152/ajpgi.2000.279.4.G757 . 11005763

[64] Philpott DJ, McKay DM, Sherman PM, Perdue MH. Infection of T84 cells with enteropathogenic Escherichia coli alters barrier and transport functions. Am J Physiol. 1996;270(4 Pt 1):G634–45. Epub 1996/04/01. 10.1152/ajpgi.1996.270.4.G634 . 8928793

[65] Hermiston ML, Gordon JI. In vivo analysis of cadherin function in the mouse intestinal epithelium: essential roles in adhesion, maintenance of differentiation, and regulation of programmed cell death. J Cell Biol. 1995;129(2):489–506. Epub 1995/04/01. ; PubMed Central PMCID: PMCPMC2199905. 7721948 10.1083/jcb.129.2.489 PMC2199905

[66] Greenbloom SL, Steinhart AH, Greenberg GR. Combination ciprofloxacin and metronidazole for active Crohn's disease. Can J Gastroenterol. 1998;12(1):53–6. Epub 1998/04/17. . 9544412 10.1155/1998/349460

[67] Olsan EE, Byndloss MX, Faber F, Rivera-Chavez F, Tsolis RM, Baumler AJ. Colonization resistance: The deconvolution of a complex trait. J Biol Chem. 2017;292(21):8577–81. Epub 2017/04/09. 10.1074/jbc.R116.752295 ; PubMed Central PMCID: PMCPmc5448087. 28389556 PMC5448087

[68] Hans W, Scholmerich J, Gross V, Falk W. The role of the resident intestinal flora in acute and chronic dextran sulfate sodium-induced colitis in mice. Eur J Gastroenterol Hepatol. 2000;12(3):267–73. Epub 2000/04/06. . 10750645 10.1097/00042737-200012030-00002

[69] Ohkusa T, Yamada M, Takenaga T, Kitazume C, Yamamoto N, Sasabe M, et al [Protective effect of metronidazole in experimental ulcerative colitis induced by dextran sulfate sodium]. Nihon Shokakibyo Gakkai zasshi = The Japanese journal of gastro-enterology. 1987;84(10):2337–46. Epub 1987/10/01. . 2449552

[70] Khalil PN, Weiler V, Nelson PJ, Khalil MN, Moosmann S, Mutschler WE, et al Nonmyeloablative stem cell therapy enhances microcirculation and tissue regeneration in murine inflammatory bowel disease. Gastroenterology. 2007;132(3):944–54. Epub 2007/03/27. 10.1053/j.gastro.2006.12.029 . 17383423

[71] Gonzalez MA, Gonzalez-Rey E, Rico L, Buscher D, Delgado M. Adipose-derived mesenchymal stem cells alleviate experimental colitis by inhibiting inflammatory and autoimmune responses. Gastroenterology. 2009;136(3):978–89. Epub 2009/01/13. 10.1053/j.gastro.2008.11.041 . 19135996

[72] Zhou Q, Price DD, Dreher KL, Pronold B, Callam CS, Sharma J, et al Localized colonic stem cell transplantation enhances tissue regeneration in murine colitis. J Cell Mol Med. 2012;16(8):1900–15. Epub 2011/11/05. 10.1111/j.1582-4934.2011.01485.x ; PubMed Central PMCID: PMCPmc3822701. 22050903 PMC3822701

